# Optimization of RNAi efficiency in PVD neuron of *C*. *elegans*

**DOI:** 10.1371/journal.pone.0298766

**Published:** 2024-03-18

**Authors:** Pallavi Singh, Kavinila Selvarasu, Anindya Ghosh-Roy

**Affiliations:** Department of Cellular & Molecular Neuroscience, National Brain Research Centre, Manesar, Haryana, India; INSERM U869, FRANCE

## Abstract

PVD neuron of *C*. *elegans* has become an attractive model for the study of dendrite development and regeneration due to its elaborate and stereotype dendrite morphology. RNA interference (RNAi) by feeding *E*. *coli* expressing dsRNA has been the basis of several genome wide screens performed using *C*. *elegans*. However, the feeding method often fails when it comes to knocking down genes in nervous system. In order to optimize the RNAi conditions for PVD neuron, we fed the worm strains with *E*. *coli* HT115 bacteria expressing dsRNA against *mec-3*, *hpo-30*, and *tiam-1*, whose loss of function are known to show dendrite morphology defects in PVD neuron. We found that RNAi of these genes in the available sensitive backgrounds including the one expresses *sid-1* under unc-*119* promoter, although resulted in reduction of dendrite branching, the phenotypes were significantly modest compared to the respective loss of function mutants. In order to enhance RNAi in PVD neurons, we generated a strain that expressed *sid-1* under the promoter *mec-3*, which exhibits strong expression in PVD. When *Pmec-3*::*sid-1* is expressed in either *nre-1(-)lin-15b(-)* or *lin-15b(-)* backgrounds, the higher order branching phenotype after RNAi of *mec-3*, *hpo-30*, and *tiam-1* was significantly enhanced as compared to the genetic background alone. Moreover, knockdown of genes playing role in dendrite regeneration in the *nre-1(-)lin-15b(-)*, P*mec-3-sid-1[+]* background resulted in significant reduction in dendrite regeneration following laser injury. The extent of dendrite regrowth due to the RNAi of *aff-1* or *ced-10* in our optimized strain was comparable to that of *aff-1* and *ced-10* mutants. Essentially, our strain expressing *sid-1* in PVD neuron, provides an RNAi optimized platform for high throughput screening of genes involved in PVD development, maintenance and regeneration.

## Introduction

The PVD neuron in *C*. *elegans* serves as an excellent model to understand the molecular basis of neuronal development and function [1]. This neuron helps worm to process multiple sensory functions including harsh touch sensation and proprioception [2]. Many concepts for neuronal development have been understood using PVD neuron as a model [3, 4]. PVD neuron displays a stereotypic structure characterized by an orthogonal array of dendritic branches that span a significant portion of worm’s body [1, 5]. The higher order branches are arranged in a menorah like fashion [6] and the tertiary branches, from any given menorah and the adjacent menorahs, establish self-avoidance to prevent physical contact, facilitated by netrin *unc-40* signaling [7–9]. *C*. *elegans* specific fusogens AFF-1 and EFF-1 sculpt this elaborate dendritic architecture of PVD neuron [6]. The guidance cue receptors and the F-actin cytoskeleton machineries collaborate to promote the extension of these branches [10, 11]. Furthermore, The quaternary branches are stabilized by the physical interactions with the epidermis and muscle through a molecular repertoire represented by these tissues [12, 13].

The higher order branches of PVD undergo dynamic modulation in response to mechanical stimuli from the environment [14]. Additionally, these dendrites display degeneration like phenomenon in old animals due to abnormal expression of immunological peptides [15]. However, the molecular mechanisms governing these processes are poorly defined. It has also been seen that upon laser mediated injury, the primary dendrites exhibit various regeneration phenomena, including self-fusion between proximal and distal dendrites, branching, and regrowth [16–18]. The self-fusion process is dependent on epidermal secretion of AFF-1 fusogen [17], while regrowth and branching rely on neuron specific function of CED-10 RAC GTPase [18]. Recent reports have also described dendrite regeneration phenomena in both fly and vertebrate model systems [19–21].

A high throughput screening of conserved molecular pathways governing the dendrite regeneration and degeneration processes in PVD neuron might yield relevant insights into these processes. Since its discovery in the late 1990s, RNAi has been a powerful tool for gene silencing and functional genomic studies in *C*. *elegans* [22–24]. This tool helps in an unbiased identification of the molecular pathways controlling a given biological process in worm system [25–27]. However, it is often difficult to get an effective knockdown of genes in the neurons using the traditional feeding method [28]. In order to overcome these limitations, researchers have identified various RNAi sensitive strains which exhibit an increased responsiveness to RNAi [29–32]. For example, mutation in the tumor suppressor gene *retinoblastoma (rb)/lin-15b* enhances the p-granule formation in somatic cells, thereby promoting RNAi mediated gene silencing [31, 33]. Several reports have shown that tissue-specific over-expression of the transmembrane protein SID-1, which is a channel for transporting dsRNA, greatly facilitates the uptake of dsRNA by that tissue [34–37]. This approach has been used to design strains sensitive for RNAi specifically in the motor neurons and touch neurons of *C*. *elegans* [34, 38].

While there have been a few studies which utilize RNAi to knockdown candidate genes in PVD neurons, the penetrance of the associated neuronal phenotypes observed in these studies seems to be modest [7, 39, 40]. This suggests that there might be a scope to enhance the efficacy of RNAi in PVD neurons through additional modifications to the existing RNAi sensitive strains. In this study, we systematically analyzed the effectiveness of RNAi in these sensitized backgrounds by assessing the loss of function phenotype of the genes known to regulate PVD dendrite morphology, such as *mec-3*, *hpo-30* and *tiam-1*. We observed that the penetrance of the RNAi induced phenotype, for any of these genes in *sid-1(-); lin-15b(-);Punc-119*::*sid-1[+]* background is similar to the general sensitive background such as *lin-1b(-)* or *nre-1(-)lin-15b(-)*.To improve the knockdown of these genes in PVD neurons specifically, we constructed a strain expressing the dsRNA channel SID-1 under the *mec-3* promoter, which exhibits strong expression in PVD neurons. We found that this *Pmec-3*::*sid-1* expression combined with RNAi sensitivity in either *lin-15b(-)* or *nre-1(-)lin-15b(-)* background, significantly enhances the penetrance of PVD neuron phenotypes associated with the knockdown of *mec-3*, *hpo-30* and *tiam-1* compared to the penetrance seen in *lin-15b(-)* or *nre-1(-) lin-15b(-)* backgrounds alone. We further showed that the extent of dendrite regeneration observed on RNAi mediated knockdown of *ced-10* and *aff-1* genes in *nre-1(-) lin-15b(-)*; P*mec-3*:: *sid-1[+]* background is comparable to the loss of function mutant alleles of these genes. This illustrates that combining the general RNAi sensitivity of the *nre-1(-) lin-15b(-)* background with the PVD specific increase in *sid-1* expression optimizes RNAi efficiency in PVD neurons.

## Results

### RNAi mediated knockdown of PVD-specific genes in the existing RNAi sensitive backgrounds show mild phenotypes

The primary goal of this study was to identify a genetic background that can efficiently knockdown candidate genes in PVD neurons. In order to achieve this, we first aimed to optimize the conditions for induction of ds RNA production in the *E*. *coli* HT115 bacteria. This step was essential because different studies have used a wide range of induction conditions in their experiments [23, 38, 41]. In our initial experiments we assessed the efficacy of knocking down of some ubiquitously expressing genes such as *dhc-1* (dynein-heavy chain), *unc-22* and *gbp-1* in the wild-type N2 Bristol strain[41]. In our hands, the induction of primary culture (condition-1) produced stronger phenotypes associated with the knockdown of these and a few other globally expressing genes in wild type strain compared to the induction of secondary culture (condition-2) (S1A Fig). Therefore, we chose condition-1 to pursue RNAi for genes related to the nervous system (S1C Fig). Our RNAi experiments on *unc-14*, *unc-13*, *snb-1*, *unc-31*, and *unc-25* revealed that both the *lin-15b(-)* and *sid-1(-); lin-15b(-); Punc-119*::*sid-1(+)* strains produce consistent loss of function phenotypes of these neuronal genes (S1C Fig).

**Fig 1:**
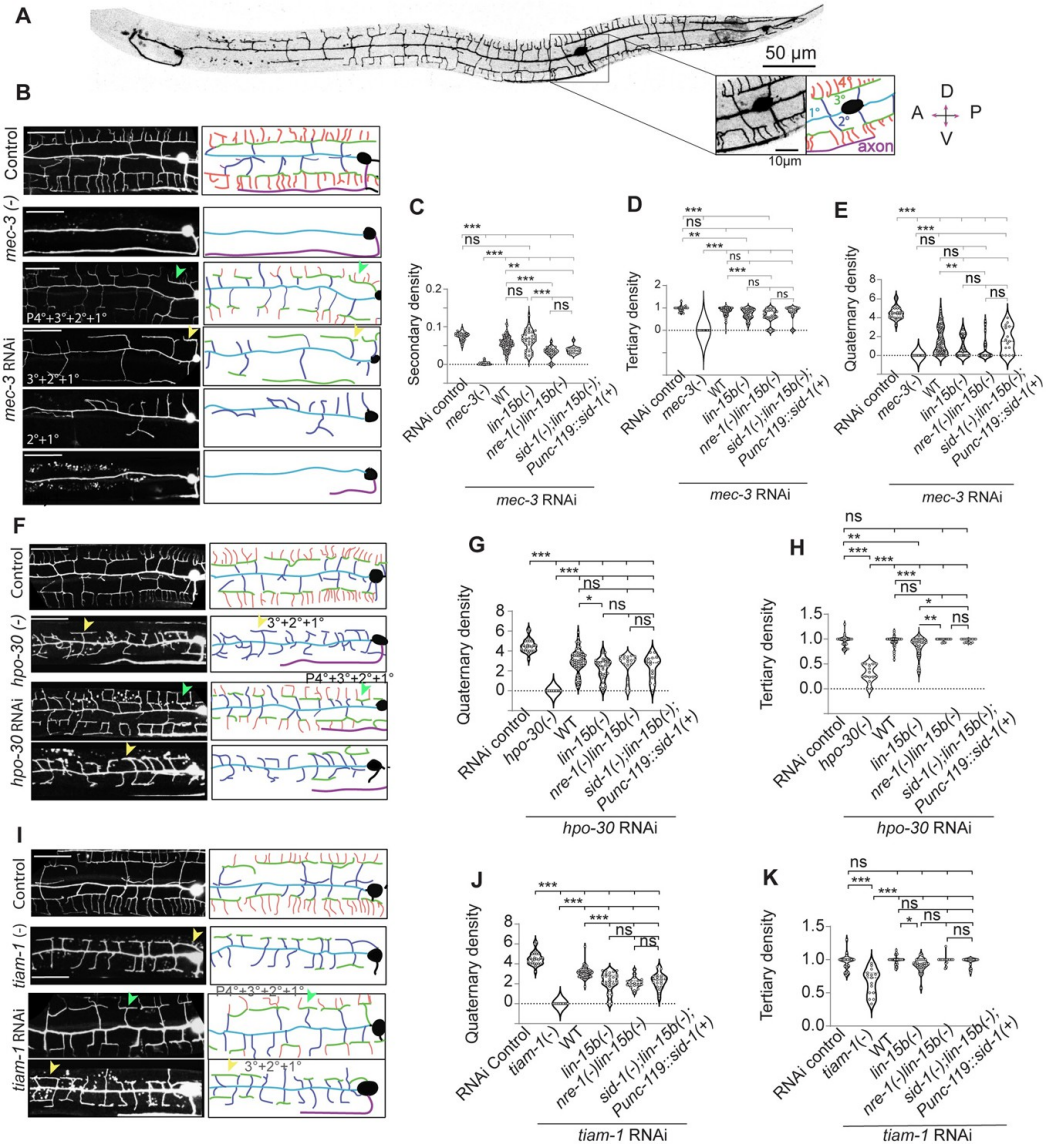
Standard sensitive strains cannot efficiently knockdown genes in PVD to cause dendritic phenotype close to the respective loss of function mutant. A) The stitched confocal image of PVD neuron expressing GFP-reporter *wdIs52 [pF49H12*.*4*:: *GFP]* shows the elaborate dendritic branches of this neuron. The magnified inset and its schematic indicate the PVD soma, hierarchy of dendritic branches (1°/primary, 2°/secondary, 3°/Tertiary, and 4°/ quaternary) and its ventral axon. (B), (F), (I) Confocal images (left) and its schematic tracings (right) show the range of phenotype for dendritic branches shown in different colors, caused due to RNAi against PVD-specific genes such as *mec-3*, *hpo-30*, *tiam-1* in various sensitive background. The L4440 bacteria was used as RNAi control. The images of the respective loss of function mutants are also presented. For example, *mec-3(-)* represents the loss of function mutant for *mec-3*. The yellow arrows show the tertiary branch without any quaternary (3 ^o^ +2 ^o^ +1 ^o^). Similarly, the green arrows show the tertiary branches with partial presence of quaternary (P4 ^o^ +3 ^o^ +2 ^o^ +1 ^o^.) The plots (C-E) shows the density of secondary, tertiary and quaternary dendrite to give the quantitative description of *mec-3* RNAi and mutant phenotype. The plots (G-H) & (J-K) show the density of quaternary and tertiary dendrite due to the RNAi of *hpo-30* and *tiam-1*. Scale bar is 25 μm for all the images in (B),(F),(I). Statistics: For (C-E),(G-H),(J-K) One-way ANOVA with Tukey’s multiple comparison test, and number of worms (n), Biological replicates (N) are 15≤n≤81, 1≤N≤4, p<0.033*, 0.002**, 0.001***, ns, not significant.

We next tested whether any of these strains can be used for effective knockdown of genes known to shape the architecture of PVD dendrites [5], thereby producing relevant phenotypes. The dendrites in PVD neuron span across the whole body (Fig 1A) and show an orthogonal pattern in higher order branches (1^o^, 2^o^, 3^o^ & 4^o,^ in Fig 1A). We selected *mec-3*, *hpo-30* and *tiam-1* which are required cell autonomously for the formation of these higher order branches in PVD [5, 42, 43]. We performed RNAi of *mec-3* in *lin-15b(-)*, *nre-1(-)lin-15b(-)* and *sid-1(-); lin15b(-); Punc-119*::*sid-1(+)* backgrounds and compared the dendritic arborization with the null mutant of *mec-3* (Fig 1C–1E). In *mec-3 (e1338)* mutant, the secondary/primary ratio per unit length of primary is close to zero as there is no secondary branch in this mutant (Fig 1B and 1C) as seen before [43]. Whereas the RNAi of *mec-3* in the sensitive backgrounds caused a wide range of phenotypes (Fig 1B). Some animals had partial quaternary (P4) or complete absence of quaternary (P4 ^o^ +3 ^o^ +2 ^o^ +1 ^o^ or 3 ^o^ +2 ^o^ +1 ^o^ in Fig 1B), while in some, both quaternary and tertiary branches were missing (2 ^o^ +1 ^o^ in Fig 1B). In the rest of the population, the phenotype was like *mec-3* null mutant, where all the higher order branches were missing (Fig 1B). Therefore, the phenotype related to the secondary/primary ratio in the sensitive backgrounds due to the RNAi of *mec-3* gene was significantly weaker compared to the null mutant (Fig 1C). Likewise, both the quaternary/tertiary and tertiary/secondary ratios were significantly reduced in the sensitive backgrounds *lin-15b(-)* & *nre-1(-)lin-15b(-)* upon knockdown of *mec-3* gene (Fig 1D and 1E).

The RNAi of many other genes that are known to regulate PVD neuron morphology [5, 7] produced significantly penetrant neuronal phenotypes in *nre-1(-)lin-15b(-)* background using ‘condition-I’ for dsRNA induction in *E*. *coli* HT115 bacteria (S2 Fig). Similarly, we tested the knockdown of genes like *hpo-30*, and *tiam-1* which are required for the formation of the quaternary branches [10, 44]. In the loss of function mutant of *hpo-30(ok2047)*, the quaternary/tertiary ratio is close to zero (Fig 1G) as the quaternary branches are completely missing in this mutant (yellow arrow/ Fig 1F).The knockdown of *hpo-30* using RNAi in the sensitive backgrounds also showed a reduction of quaternary branches (Fig 1F) although often some quaternary branches were still present (green arrow / Fig 1F). Therefore, the quaternary/tertiary ratio due to RNAi was significantly higher compared to the mutant (P<0.001,*** One-way ANOVA with Tukey’s multiple comparison test, Fig 1G). Similar trend was also noticed when we compared the quaternary branch phenotype in the loss of function mutant of *tiam-1(ok772)* to the RNAi mediated knockdown of the same gene in the sensitive backgrounds (Fig 1I and 1J). However, we found that the strength of the phenotypes were comparable in *lin-15b(-)*, *nre-1(-)lin-15b(-)* and *sid-1(-); lin-15b(-); Punc-119*::*sid-1(+)* strains (Fig 1C–1E, 1G–1H, 1J and 1K). We initially expected that *Punc-119*::*sid-1(+)* strain would cause significantly stronger penetrance of PVD specific phenotype as compared to the loss of either *lin-15b*(-) or *nre-1(-)lin-15b(-)* alone.

These observations suggested that RNAi mediated knockdown of the candidate genes in the PVD neuron using available general and neuron specific RNAi sensitive strains is not as efficient as the loss of function mutations of respective genes. And this leaves a window open for further optimization of RNAi in PVD neurons to enhance its efficiency by making modifications to existing RNAi sensitive strains.

### PVD specific expression of *sid-1* helped knockdown of genes in PVD neuron

**Fig 2:**
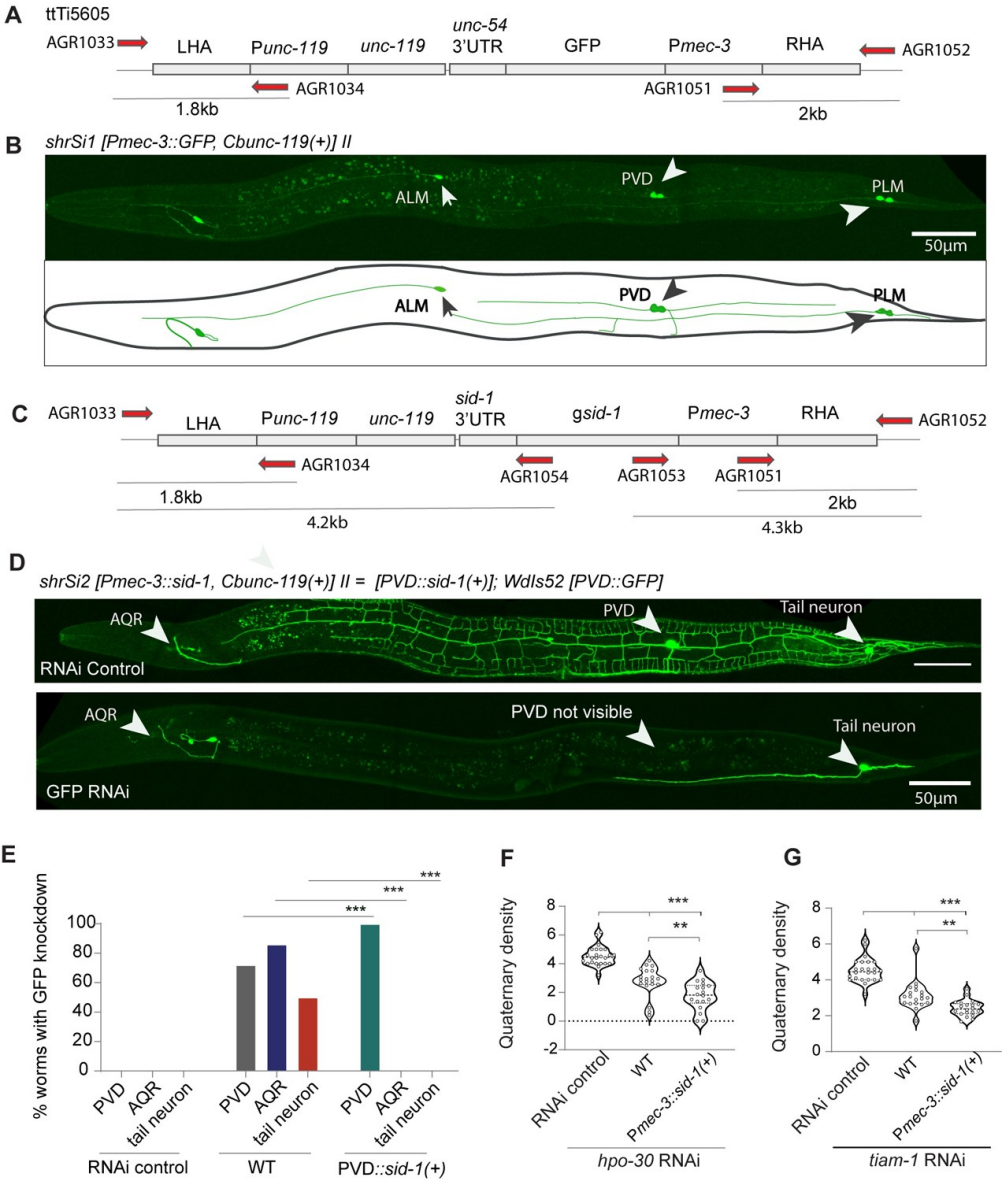
PVD specific expression of *sid-1* helps PVD-specific reduction of target-genes using RNAi. (A) The illustration of P*mec-3*::*GFP* cassette inserted in the locus harbouring MosI element ttTi5605 on chromosome-II. LHA & RHA denote Left Homology Arm and Right Homology Arm. (B) The confocal image and illustration showing the expression pattern of single copy inserted transgene *shrSI1*[*Pmec-3*::GFP]. The white arrowheads are marking GFP expression in PVD, ALM, and PLM neuron. (C) The illustration for the single copy insertion *shrSI2 [Pmec-3*::*sid-1]* driving *sid-1* in PVD neuron. (D) The confocal images of PVD neuron expressing *wdIs52 [pF49H12*.*4*::*GFP]* after feeding the worms with L4440 (RNAi Control) and GFP RNAi bacteria in *shrSI2 [Pmec-3*::*sid-1]* background. It shows specific reduction of GFP in PVD. White arrowheads shows three neurons AQR, PVD, Tail neuron visible in the *wdIs52 [pF49H12*.*4*::*GFP]* reporter strain. (E) Percentage of worms with GFP knockdown in these three neurons are plotted separately for each genotypes showing PVD::*sid-1*(*Pmec-3*::*sid-1)* cassette effectively knocking down GFP in PVD neuron specifically. (F-G) Graph shows quaternary density for *hpo-30* and *tiam-1* RNAi fed worms was significantly reduced in *Pmec-3*::*sid-1(+)* strain. Statistics, For E, Fisher’s exact test 10≤n≤15, 1≤N≤2, (F-G) One-way ANOVA with Tukey’s multiple comparison test, 18≤n≤24, 1≤N≤2 p<0.033*, 0.002**, 0.001***, ns, not significant. N = number of independent replicates, and n = number of worms tested.

To enhance the phenotype associated to the RNAi of PVD-specific genes, we thought of expressing the dsRNA channel SID-1 using PVD specific promoter [35, 36]. The cell-specific expression of *sid-1* in touch or motor neuron enhanced the phenotypes related to the knockdown of genes in these neuron [34, 38]. We screened for a promoter that strongly expresses in PVD neuron using the neuronal cell atlas (CENGEN) [45, 46]. We found that the expression of *unc-119* gene in PVD neuron (103.59 Transcripts per million/TPM) is relatively lower as compared to the expression level *mec-3* in same neuron (4067.391 TPM, CENGEN) [45]. Additionally, *mec-3* is expressed early in PVD, starting from early larval stages [7], allowing RNAi to begin early for targeted genes. Therefore, we generated an integrated strain that expresses *sid-1* under the *mec-3* promoter to test if it enhances RNAi in the PVD neurons. We made a single copy insertion of P*mec-3*::*sid-1*[+] (Fig 2A–2C) by the mobilization of Mos1 transposon element [47, 48]. As a control experiment we expressed P*mec-3*::GFP (Fig 2A) using the same single copy insertion transgenic method [47]. The strain expressing P*mec-3*::GFP showed GFP expression in PVD (Arrowhead, Fig 2B) as well as in the PLM and ALM neurons (Fig 2B) validating the expression of *mec-3* promoter in PVD neurons. Similarly we inserted P*mec-3*::*sid-1(+)* cassette and tested this strain for enhanced RNAi by first assessing the RNAi against GFP in PVD neurons. As a control we used the transgenic strain, *wdIs52*, that expresses GFP under the promoter *pF49H12*.*4*, which is specific to PVD, AQR and the tail neuron. In the wild type *wdIs52* background, RNAi against GFP causes loss of GFP expression in all these neurons(Fig 2E). However, the RNAi against GFP in the RNAi sensitive strain expressing P*mec-3*::*sid-1* caused exclusive loss of GFP reporter in PVD neuron (Fig 2D and 2E), confirming that effect of *sid-1* is specific to PVD neuron. In 100% of the worms, in P*mec-3*::*sid-1* background, GFP was not visible in PVD neuron (Fig 2E). Further, to test for PVD specific phenotypic defects in the *Pmec-3*::*sid-1* strain, we knocked down *hpo-30 and tiam-1* using RNAi, We observed a significant reduction in density of quaternary branches as compared to knockdown in the wild-type worms (Fig 2F and 2G), indicating the PVD specific effectiveness of the *sid-1* expression in the strain we developed.

### RNAi efficiency in PVD neuron can be synergistically enhanced by PVD specific expression of *sid-1* and sensitivity due to loss of *lin-15b*

**Fig 3:**
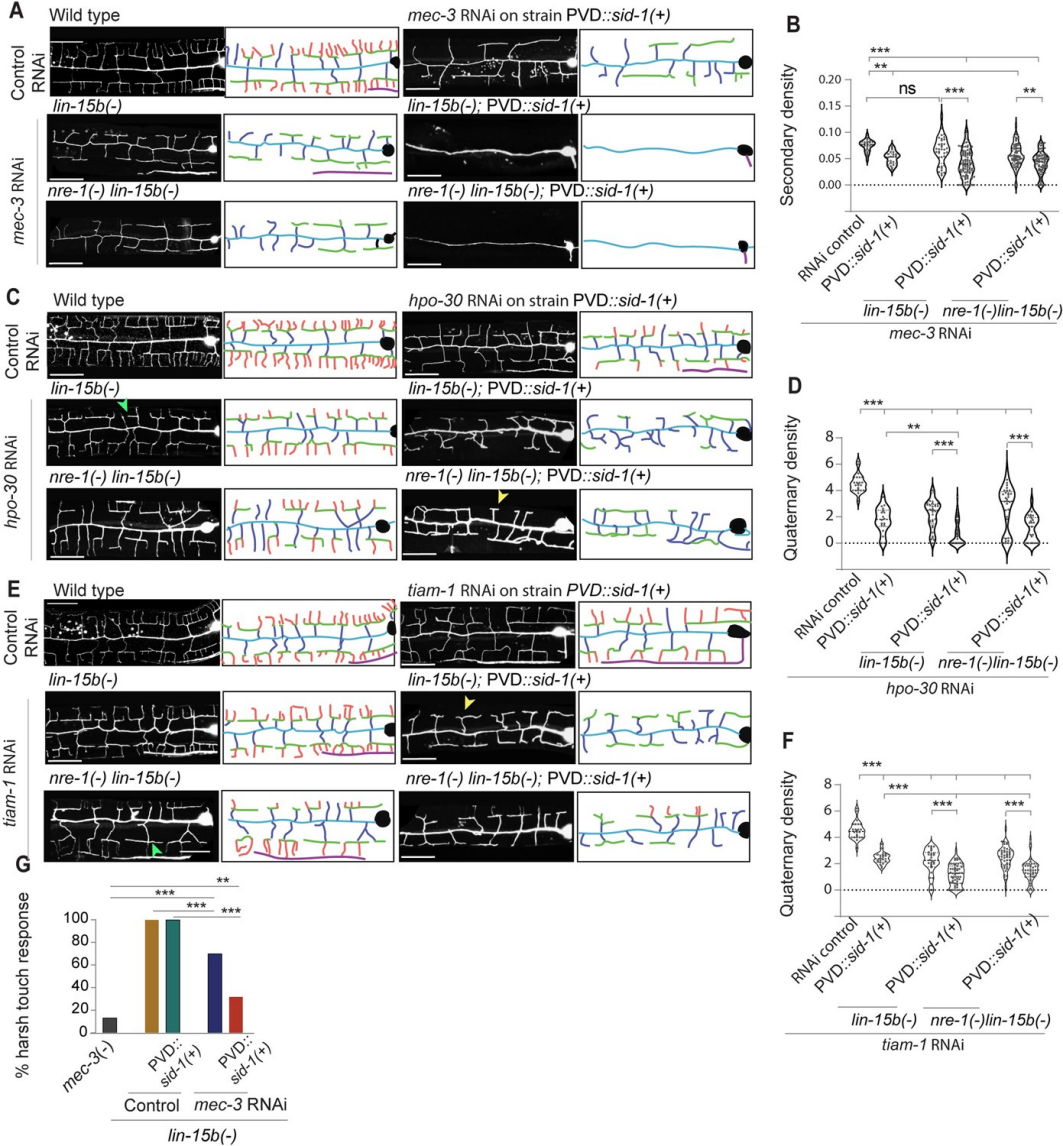
PVD specific expression of *sid-1* enhances the penetrance of RNAi phenotype. (A, C & E) The confocal images (left) and respective illustrations (right) show the RNAi phenotypes of *mec-3*, *hpo-30 or tiam-1* in standard sensitive backgrounds *lin-15b*(n744) or *nre-1(hd20) lin-15b(hd126)* and in strains with the addition of *Pmec-3*::*sid-1(+)* transgene in the similar sensitive backgrounds [*lin-15b*(n744) or *nre-1(hd20)lin-15b(hd126)]*. The yellow arrowheads mark the tertiary with its missing quaternary branches in *hpo-30 and tiam-1* RNAi image. Green arrow shows the presence of partial quaternary in *hpo-30* RNAi worms. The *Pmec-3*::*sid-1(+)* transgene is denoted as *PVD*::*sid-1(+)*. (B) The secondary density of PVD dendrite in the different genetic backgrounds with *mec-3* RNAi as shown in panel-A. An enhancement of phenotype was noticed in the strains having *PVD*::*sid-1(+)* transgene. Panel-D & F show the quaternary dendrite density in PVD upon the RNAi of *hpo-30* and *tiam-1*, respectively. (G) The percentage of worms responding to harsh touch delivered with a platinum wire in case of *mec-3* RNAi and *mec-3* mutant/*mec-3(-)*. *PVD*::*sid-1(+)*, denotes *Pmec-3*::*sid-1(+)*. The scale bar in all the images is 25μm. Statistics: For (B,D,F) One-way ANOVA with Tukey’s multiple comparison test, 24≤n≤97, 1≤N≤4, for (G) Fisher’s exact test, 10≤n≤20, 1≤N≤2 was performed, p<0.033*, 0.002**, 0.001***, ns, not significant, Biological replicates (N) and number of worms (n).

Since the RNAi of *mec-3* gene in the existing sensitive strains such as *lin-15b(-)* or *nre1(-)lin-15b(-)* did not result in strong phenotypes, we hypothesized that elevating the level of *sid-1* in these sensitive strains might enhance the phenotypes. Indeed, when we compared the secondary/primary ratio in the *nre-1(-)lin-15b(-)* and *nre1(-)lin-15b(-)*; P*mec-3*::*sid-1[+]* strains, upon knockdown of *mec-3*, we found that addition of *sid-1[+]* significantly enhanced the phenotype in the *nre-1(-)lin-15b(-)* (P< 0.002,** One-way ANOVA with Tukey’s multiple comparison test, Fig 3B). In many instances, in the *nre-1(-)lin-15b*(-); *Pmec-3*::*sid-1[+]* strain, only primary dendrite were visible (Fig 3A) resembling the phenotype seen in the *mec-3(e1338)* mutant. The addition of *sid-1[+]* in the *lin-15b(-)* strain alone also significantly reduced the secondary/primary ratio (Fig 3A and 3B). Moreover, the quaternary / tertiary ratio also significantly dropped in the *nre-1(-)lin-15b(-)*; P*mec-3*::*sid-1[+]* strain as compared to *nre-1(-)lin-15b(-)* alone (S3B Fig).

Similarly, when we compared the quaternary/tertiary ratio in the *nre-1(-)lin-15b(-)* and *nre1(-)lin-15b(-)*; P*mec-3*::*sid-1[+]* strains after RNAi of *hpo-30*, we observed that the addition of *sid-1[+]* significantly reduced the ratio in the *nre-1(-)lin-15b(-)* (P<0.001,*** One-way ANOVA with Tukey’s multiple comparison test, Fig 3C and 3D). The same trend was observed for the knockdown of *tiam-1* gene (P<0.001,*** One-way ANOVA with Tukey’s multiple comparison test, Fig 3E and 3F), which is exclusively required for the stabilization of the quaternary branches in PVD [10]. Moreover, the P*mec-3*::*sid-1 [+]* background was significantly efficient as compared to the P*unc-119*::*sid-1[+]* in perturbing the formation of quaternary branches upon knockdown of *tiam-1* or *hpo-30* gene (S3H and S3I Fig).

Furthermore, we observed that the harsh touch response was significantly reduced upon RNAi of *mec-3* in the *lin-15b(-)*, *Pmec-3*::*sid-1[+]* background as compared to the *lin-15b(-)* single mutant (Fig 3G). The touch response in the *lin-15b(-)*, *Pmec-3*::*sid-1[+]* background was close to the response observed in *mec-3* mutant [49].

Therefore, in both *lin-15b(-)*, *Pmec-3*::*sid-1[+]* and *nre1(-) lin-15b(-)*; *Pmec-3*::*sid-1[+]* strains, we were able to achieve a synergistic effect of the *lin-15b(-)* mutation and PVD specific expression of *sid-1* for the knockdown effect of PVD specific genes.

### The genes controlling dendrite regeneration pathways can be knocked down by RNAi in *nre1(-)lin-15b(-)*; *Pmec-3*::*sid-1[+]* background

**Fig 4:**
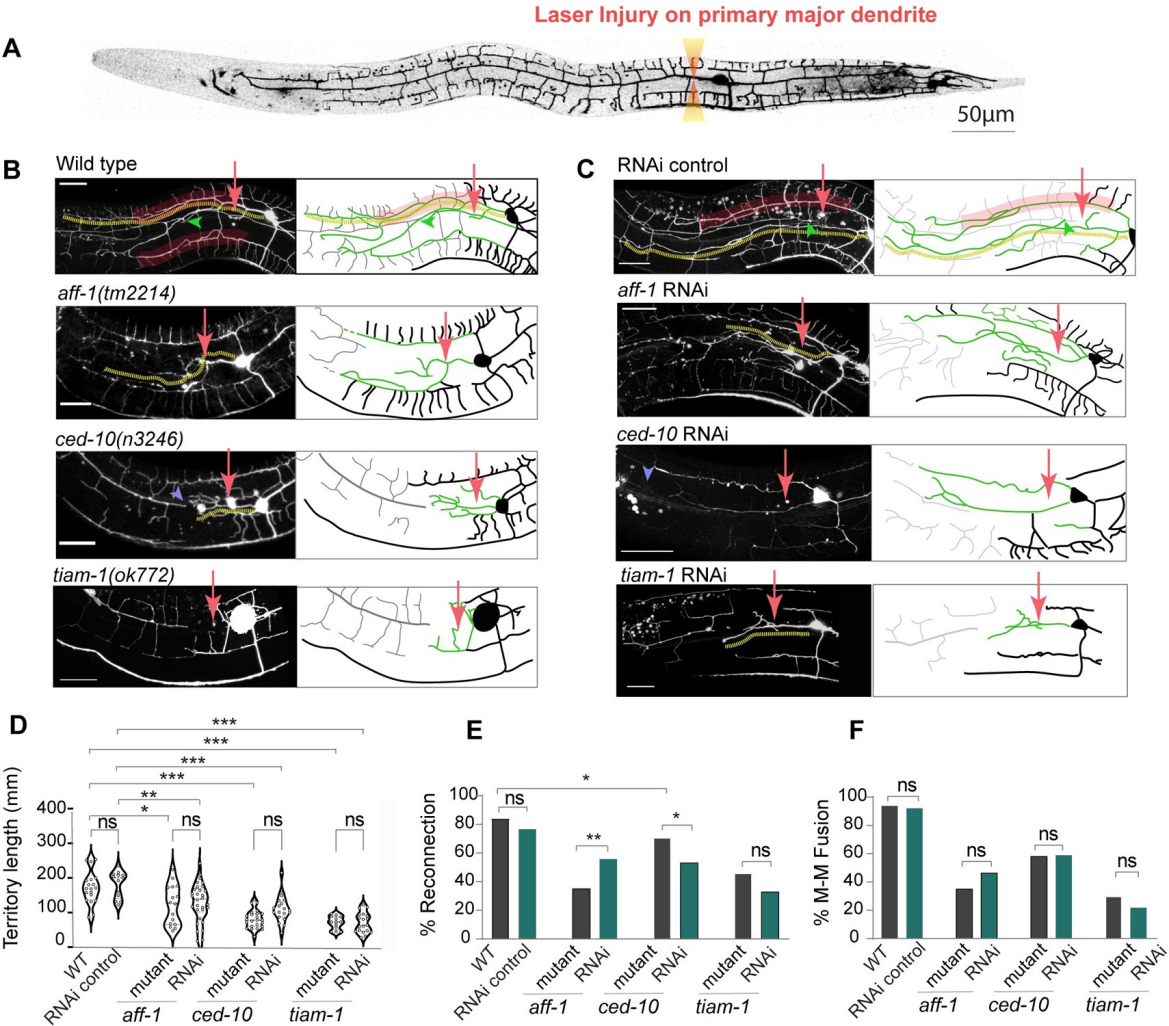
RNAi of genes in *sid-1* expressing strain and respective loss of function mutants cause comparable dendrite regeneration phenotype in PVD. (A) Stitched confocal image of whole PVD showing the site of Laser injury in the primary major dendrite (B) Confocal images of regeneration pattern at 24 hours post-dendrotomy in Wildtype (control) and mutant worms. (C) Images of regenerating PVD dendrite after the RNAi performed in *Pmec-3*::*sid-1[+]; nre-1(-)lin-15b(-)* RNAi worms. In (B-C) confocal images(left) and schematics(right), menorah-menorah fusion is highlighted in red box, reconnection is marked with green arrowhead, yellow dotted line shows the extent of longest regenerating branch over images and schematic. Site of laser injury is marked with red arrows. Schematics on right side of these images have regrowing neurites shown in green traces, proximal PVD dendrite in black, distal portion after injury is drawn in grey, (D) Territory length i.e. length of longest neurite was measured and compared between mutants and RNAi of L4440(control), *aff-1*, *ced-10*, *tiam-1* genes in *nre-1(-)lin-15b(-)*;*Pmec-3*::*sid-1(+)* (E) Percentage of worms with reconnection events and (F) Percentage of worms with menorah-menorah fusion was compared for mutants and RNAi worms of these genes in *nre-1(-)lin-15b(-)*;*Pmec-3*::*sid-1(+)* strain. Statistics, For D, one-way ANOVA with Tukey’s multiple comparison test, 13≤n≤30, 1≤ N≤2, For (E-F) Fisher’s exact test were performed, 13≤n≤30, 1≤ N≤2, p<0.033*, 0.002**, 0.001***, ns, not significant. Scale bar for (B-C): 25μm.Biological replicates (N) and number of worms (n).

Since the RNAi mediated knockdown of *mec-3*, *tiam-1* and *hpo-30* produced strong phenotypes in *nre1(-)lin-15b(-)*; *Pmec-3*::*sid-1[+]* strain, we were encouraged to test whether one can optimally use this background in dendrite regeneration studies. Previous work has shown that the primary dendrite of PVD upon laser injury shows regeneration response [16, 18]. The dendrite regeneration depends on the RAC GTPase CED-10, GEF TIAM-1 [18] and the fusogen molecule AFF-1 [17]. Typically, following dendrotomy (Orange laser shots, Fig 4A), the primary dendrite regrows (green traces in schematic Fig 4B and 4C) and reconnects (green arrowheads) to the distal end of the injured dendrites (Fig 4B and 4C). The tertiary branches corresponding to the menorahs of proximal and distal dendrites often are fused with each other (red highlighted boxes, Fig 4B) to bypass the gap created due to the injury. This phenomenon is called menorah-menorah fusion [17]. As reported before [18], we found that the extent of regrowth after dendrotomy, indicated by ‘territory length’, is significantly reduced in the loss of function mutant of *ced-10(n3246)* (Fig 4B and 4D). Similarly, the percentage of reconnection and menorah-menorah fusion events were also significantly reduced in *ced-10* mutant (Fig 4E and 4F). Often there is a visible gap between the proximal and distal dendrites in the *ced-10* mutant (blue arrowhead, Fig 4B). When we performed RNAi of *ced-10* in *nre1(-)lin-15b(-)*; *Pmec-3*::*sid-1[+]* strain, we noticed all of these phenomena (blue arrowhead, Fig 4C), as seen in the *ced-10* mutant. There was a significant reduction in territory length, percentage of neurites showing reconnection and percentage of neurites showing menorah-menorah fusion, upon RNAi of *ced-10* (Fig 4D–4F). More interestingly, the extent of reduction in the regeneration parameters was comparable in RNAi background and *ced-10* and *tiam-1* mutant (Fig 4D–4F). Similarly, the reduction in regeneration parameters due to the RNAi of *aff-1* was also comparable to the *aff-1* mutant (Fig 4B–4F).

This suggests that the *nre-1(-) lin-15b(-);* P*mec-3*::*sid-1[+]* strain efficiently facilitates the knockdown of various genes, encompassing not only developmental branching genes but also those required in adulthood for injury-induced responses.

## Discussion

PVD neuron has been a great model to understand the development and function of nerve cells [1, 2, 6, 50]. Especially, the stereotypic and elaborate dendritic branches in PVD neuron make it an interesting system to explore the mechanism of dendrite development, maintenance, and regeneration. Combining genetics and cell biology, researchers have made progress in mechanistic understanding of how elaborate anatomy of dendrite in PVD neuron is developed [6, 10]. However, there is a big gap in our understanding of how the dendritic arbor is maintained in adulthood and how it is repaired after injury.

RNAi has been instrumental in identifying novel molecular pathways in nerve cell development [31] and neurite regeneration [51, 52] using *C*. *elegans*. However, RNAi cannot phenocopy 100% the null-mutant phenotype for various reasons including the stability and availability of target mRNA, Moreover, RNAi in nervous system is highly variable depending on neuron type [53] and genes being targeted. Researchers have always had to try various sensitive strains in order to get effective knockdown of genes in neurons [7, 31]. The mutations in the genes such as *lin-15b*, *nre-1* and *eri-1*, which negatively regulate RNAi process, are often used to enhance the phenotypes in neuron [7, 31–33]. The lack of expression of the dsRNA channel SID-1 in the nervous system makes systemic RNAi inefficient in neuron [34]. To overcome this challenge, researchers often mis-expressed *sid-1* in neurons to enhance the RNAi efficiency [34, 38]. The existing strain that overexpresses *sid-1* under pan-neuronal promoter P*unc-119* did not give effective phenotype as compared to *nre-1(-)lin-15b(-)* or *lin-15b(-)* mutant alone for PVD specific phenotypes. Therefore, we ventured our effort to try stronger PVD-specific promoter for obtaining stronger phenotype. Our results with the knockdown of *mec-3*, *hpo-30* and *tiam-1* genes indicated that indeed addition of P*mec-3*::*sid-1* enhances the associated dendrite branching phenotypes in both the *lin-15b(-)* and *nre-1(-)lin-15b(-)* backgrounds. The *nre-1(-) lin-15b(-) Pmec-3*::*sid-1(+)* background also allows us to achieve the expected phenotype for dendrite regeneration following laser injury through the RNAi of *ced-10* and *aff-1*. Therefore, this will be a highly useful tool for the researchers to study the questions related to PVD neuron.

## Materials and methods

### *C*. *elegans* strains and genetics

In this study, *C*. *elegans* strains were maintained at 20°C on *E*. *coli* OP50 bacterial lawn seeded over Nematode Growth Medium (NGM) plates [54]. The loss of function mutation is represented as (-). For example, loss of function allele of *mec-3 (e1338)* represented as *mec-3(-)*. The mutants used in this study are mostly loss of function by deletion or substitution unless otherwise mentioned. These mutants were obtained from Caenorhabditis Genetics Centre (CGC). The mutations crossed with *wdIs52 [pF49H12*.*4*:: *GFP]* strain carrying PVD specific GFP marker to aid visualization and microscopy and genotyped using their respective primers. Details of strain used for the study is provided in S1 Table

### Optimization of the induction of ds RNA expression

To optimize the induction of dsRNA expression in *E*. *coli* HT115 bacteria in our hand, we tried three different induction conditions suggested in previous reports [23, 38, 41] with some modification i.e. condition 1 (primary culture), condition 2 (secondary culture with IPTG induction), condition 3 (secondary culture without IPTG induction). After seeding the bacteria grown under each condition, L4 staged worms (5–10 worms) were transferred onto these NGM plates containing carbenicillin, tetracycline, IPTG and was allowed to grow and give progenies to conduct experiments. The bacteria expressing various dsRNA were obtained from Arhinger’s and Vidal’s Library [55, 56] (S2 Table).

### Condition I

The *E*.*coli* HT115 bacteria carrying RNAi clones targeting specific genes were thawed from -80 deg and grown in Luria-Bertani (LB)-plates with 50 ug/ml carbenicillin and 12.5 ug/ml tetracycline and inoculated at 37°C. Then a single colony was inoculated and grown at 37°C in 4 ml LB in an incubator-shaker till it reached OD600 of 0.8. This primary culture was then pelleted and resuspended in 1X M9 buffer supplemented with 1.5 mM IPTG, carbenicillin and tetracycline. The resuspended culture was seeded onto NGM pates containing same concentration of carbenicillin, tetracycline, and IPTG. These plates were prepared two days in advance. Seeded plates were incubated at 25°C for a duration of 36 hours for the induction of the RNAi construct within the bacteria. The condition-I involving the induction of primary culture was used before [41].

### Condition II

The primary culture was grown in LB medium containing 50 ug/ml carbenicillin and 12.5 ug/ml tetracycline at 37°C overnight as described in ‘condition I’. The overnight grown primary culture then used to set secondary culture at 1 in 4 dilution in LB media containing carbenicillin, tetracycline, and 1Mm IPTG. The secondary culture was kept at 37°C incubator-shaker until it reached an OD600 of 0.5–0.6. Subsequently the bacteria was pelleted down and resuspended in LB medium containing IPTG, carbenicillin, and tetracycline. Resuspended bacteria was seeded onto NGM plates containing antibiotics and IPTG. The plates were kept for induction period of 8 hours at 25°C.

### Condition III

Same steps were performed as in Condition II with few modifications such as IPTG induction in secondary culture was not done and bacteria were grown until it reached OD600 of 0.5–0.6then, seeded plates were allowed to grow for 48hrs at room temperature as done before [38].

### RNAi using optimized condition

First optimal condition for induction of RNAi in *E*. *coli* was determined by performing RNAi against genes whose knockdown is known to produce strong phenotypes such as failure embryo-hatching, sterility or twitching of muscle etc. in N2 wild type strain. For example, we tested *dhc-1*, *gpb-1*, *unc-22* under three mentioned conditions in N2 background (S1A Fig). In our hand, the Condition-I produced stronger phenotypes as compared to the Condition-II and Condition-III (S1A Fig). Few other genes were also tested for further confirmation of the efficiency of ‘Condition-I’, such as *par-1*, *par-3*, *skn-1*, *dnc-1*, *bir-1*, *pal-1*, *plk-1*, *ama-1*, which are ubiquitously required in worm and RNAi of these genes produce global phenotype [41]. RNAi of many of the tested genes resulted in 100% penetrance in the wild type N2 Bristol strain (S1B Fig). Using this optimal induction condition (condition I), we performed RNAi of genes such as *unc-14*, *unc-13*, *snb-1*, *unc-31*, *unc-25* that are pan-neuronally required [34, 38], We used the strains that are shown to enhance RNAi sensitivity (S1C Fig). For each respective gene (S1A–S1C Fig), ten P0s were fed RNAi and their progenies were scored after 3 days for phenotype. Therefore, the percentage of phenotypes was calculated from 300–350 progenies for phenotypes like uncoordinated, paralyzed, shrinker, and twitching.

### MOS1 element-related single copy insertion to construct P*mec-3*::*sid-1* transgenic strain

Mos1 transposon element-related method was used to insert P*mec-3*::*sid-1* in single copy as described before [47, 48]. The P*mec-3*::*sid-1*+*unc-119* (pNBR65) was cloned in pcfj150 targeting vector [47] (Fig 2A). The genomic *sid-1* was amplified from *sid-1* plasmid (TU866) [34] using AGR1016 and AGR1017 primers. The pcfj150 backbone was amplified using AGR 1012 and AGR1013 primers and *mec-3* promoter was amplified with primers AGR1014 and AGR1015. These three fragments were assembled into a plasmid using In-Fusion reaction (cloning primer details in S3 Table). For the confirmation of expression pattern of *mec-3* promoter, *Pmec-3*::*GFP+unc-119* (pNBR64 cassette was made (Fig 2A).

The uncoordinated progenies of EG6699 [ttTi5605 II; *unc-119(ed9)*III; oxEx1578 [eft-3p::GFP+ Cbr-unc-119(+)] strain were injected with injection mix containing plasmids 50ng/μl of Pcfj601(P*eft-3*::transposase), 50ng/ul of cassette *Pmec-3*::*sid-1+unc-119*, 29ng/ul Pma122 (Phsp::*peel-1*) along with marker plasmids i.e. 10ng/ul Pgh8 (*Prab-3*::mcherry), 2.5ng/ul pcfj90 (P*myo-2*::mcherry), 5ng/ul pcfj104 (*Pmyo-3*::mcherry), 20ng/ul 100 bp ladder. First, for confirmation of expression pattern of *mec-3* promoter, *Pmec-3*::GFP+*unc-119* cassette was inserted using same strategy [48]. Around 60–70 worms were injected, and two worms were kept in each plate. Plates were kept at 25°C for a week till starved. Then for 3 hours it was kept at 34°C for *Peel-1* mediated negative selection, worms with extrachromosomal array can’t survive this heat shock. Then, these plates were transferred to 20°C for a day, 10 healthy L2-L3 non-unc worms were chosen from plates and genotyped using PCR, made homozygous for *Pmec-3*::*sid-1*(+) and outcrossed. For confirmation of insertion two PCR of 1.8kb and 2kb was done using primers (AGR1033 & AGR1034, S3 Table) on upstream of ttTi5605 and Punc-119 respectively (Fig 2). Other set of primers (AGR1051 & AGR1052, S3 Table) were designed on other end i.e., downstream to ttTi5605 and *Pmec-3* (Fig 2). For confirmation of full *Pmec-3*::*sid-1* cassette insertion, long PCR of 4.2kb and 4.3kb using primers AGR1053 and AGR1054 was also performed (Fig 2). PCR primer details are given in S3 Table. After confirmation by PCR and outcrossing, we used *shrSI1* [*Pmec-3*::GFP*+unc-119*] and *shrSI2* [*Pmec-3*::*sid-1* + *unc-119*], for our experiments as shown in Figs 2–4.

### Imaging of PVD neuron

The worms were mounted in 10 mM Levamisole hydrochloride (Sigma®) solution on the 5% agarose (Sigma®) pads made on the glass-slides. The worms were imaged with 63X/1.4NA oil objective of Nikon® A1R confocal system at a voxel resolution of 0.41μm x 0.41μm x 1μm and tile imaging module using imaging lasers 488nm (GFP), 543nm (mCherry/RFP) with 1–1.8 AU pinhole at 512x512 pixel resolution files for further analysis. For regeneration study, images were obtained at 24 hrs post-injury using same imaging condition.

### Dendrite branch quantification

PVD dendrite branch density was quantified as quaternary density, tertiary density and secondary density to normalize the phenotypes acquired in images (Fig 1B and 1F and 1I) encompassing from cell body till the middle of major dendrite using following formula: Quaternary density: Total number of quaternary dendrites / Total number of tertiary dendrites. Tertiary density: Total number of tertiary dendrites/ Total number of secondary dendrites. Secondary density: Total number of secondary dendrites / length of primary dendrite(μm). The length of primary dendrite was measured using Simple Neurite Tracer plugin in Fiji-ImageJ®.

### Laser system and dendrotomy details

Dendrotomy were conducted on worms at the L4 stage using the Bruker® ULTIMA system with spectraPhysics® Two-photon femtosecond laser. This laser is tunable and operates in the infrared range (690–1040 nm). The laser output was controlled using Conoptics pockel cells. For visualization of the PVD and injury, lasers with wavelengths of 920nm and 720nm were used simultaneously by two sets of galvanometer mirror scanning X-Y [18]. To prepare slides, worms were immobilized using Levamisole hydrochloride (10mM) on 5% agarose pads and mounted with Corning cover glass. Worm-containing slides were placed under 60X/0.9NA water objective (Olympus®) with a pixel resolution of 0.29um x 0.29um.

During the experiments (Fig 4), the PVD dendrites were severed at the first branch point, approximately 10 microns away from the cell body, using the first laser shot. This was followed by one more consecutive shot with a relative distance of 10-15um from the previous shot, resulting visible gap. After injury, worms were transferred to freshly seeded NGM plates with *E*. *coli* OP50 or RNAi bacteria for further observation.

### Dendrite regeneration quantification

Dendrite regeneration was quantified based on regrowth from site of injury and fusion related parameters like menorah-menorah fusion [16, 17] where one big menorah is supported by more than one secondary branch (Fig 4B and 4C,red highlighted box). Also we see these regrowing neurites getting connected to distal dendrite (Fig 4B and 4C, green arrowhead) evaluated as reconnection events [18]. The extent of territory covered by regrowing dendrite (Fig 4B and 4C, yellow dotted line) was measured using Simple Neurite Tracer plugin in Fiji-ImageJ® tracking the longest regenerating dendrite from cell body to the end point.

### Harsh touch behavior analysis

The worms fed on RNAi bacteria such as L4440 (empty vector/Control) and *mec-3* (dsRNA against *mec-3*) were single-selfed in 10–20 plates using eye lash pick and left for few minutes. Videos were recorded while giving them harsh touch with platinum wire posterior to vulva, recording was done for nearly 20–30 seconds as described before [2, 57]. Percentage of worms with positive response were calculated which showed observable increase in speed indicating escape response after harsh touch as represented in Fig 3G.

### Statistical analysis

The statistical analysis were performed using GraphPad Prism software (version 9.5.1). For two-sample comparisons, unpaired two tailed t-tests were used. When analyzing multiple samples, one way analysis of variance (ANOVA) was performed, followed by Tukey’s multiple comparisons test. The data used for the ANOVA analysis consisted of naturally occurring data with a normal distribution spread. To compare population data, fraction values were calculated for each sample and compared using a two-tailed chi-square Fischer’s exact contingency test.

The figure legends of the respective bar provide the information about the number of samples (n) and the number of biological replicates (N). The significance levels considered for all statistical experiments were p<0.033*, 0.002**, 0.001***. The details of statistical analysis for the all the plots associated with this study are available in S4 Table.

## Supporting information

S1 Fig Optimization of RNAi induction for ubiquitous genes and neuronal genes. (A) The percentage of phenotype involving embryonic lethality and twitching in L4440 (control), *dhc-1*, *gbp-1*, *unc-22* RNAi done in N2 Bristol background under three different induction conditions are plotted. Condition-I: induction of primary culture, Condition-II: induction of secondary culture with IPTG, and Condition-III: secondary culture was grown without IPTG induction. (B) The effectiveness of RNAi in N2 background using “condition-I” was further verified by knocking down various genes that cause embryonic lethality, twitching, larval arrest. (C) The organism-level phenotypes caused due to RNAi for genes required pan-neuronally are shown in this bar-plot. In this experiment, the RNAi was performed in the sensitive genetic backgrounds such as *eri-1(mg366)*, *lin-15b(n744)*, *nre-1(hd20)lin-15b(hd126)* and neuronal sensitive background *sid-1(pk3321); Punc-119*::*sid-1(+)* and *sid-1(pk3321); lin-15b(n744); Punc-119*::*sid-1(+)*. (A-C) 10 P0s were fed with *E*. *coli* containing dsRNA and their progenies (300–350) were scored for respective phenotype in each batch. Biological replicates (1≤N≤2). (A-C) Statistics: Fisher’s exact test were performed, p<0.033*, 0.002**, 0.001***, ns, not significant.
(TIF)

S2 Fig RNAi for various PVD neuron related genes in standard sensitive background i.e. *nre-1(hd20)lin-15b(hd126)*. (A) The images show the dendrite morphology defects in PVD neuron caused due to RNAi of various genes known to affect PVD development. The RNAi of these genes were performed in *nre-1(hd20)lin-15b(hd126)* background. The illustrations of the defects caused due to RNAi of these genes are also shown on the right. The hierarchy of PVD dendrite are shown in different colors i.e quaternary in red, tertiary in green, secondary in violet, primary in blue. Scale bar is 25 μm. (B) The RNA experiment mentioned in panel-A is summarized in a tabular form. The phenotypes associated to the RNAi of various genes are mentioned in this table. Biological replicates (1≤ N≤2).
(TIF)

S3 Fig Higher order dendrite density of PVD neuron upon *mec-3*, *hpo-30* and *tiam-1* RNAi in various RNAi sensitive strain. (A-B) Tertiary and quaternary density of PVD dendrites for *mec-3* RNAi in different sensitive backgrounds. (C-D) represents secondary and tertiary density of PVD dendrites for *hpo-30* RNAi worms. Similarly, (E-F) show the tertiary and secondary density for *tiam-1* RNAi. Biological replicates (1≤N≤4) and number of worms (24≤n≤97). Statistics: One-way ANOVA with Tukey’s multiple comparison test p<0.033*, 0.002**, 0.001***, ns (not significant). (G-I) shows the comparative analysis of phenotypic penetrance involving PVD higher order branching in strains expressing *sid-1* under *unc-119* and *mec-3* promoter. (G) shows the secondary density in strains i.e. *sid-1(-); lin-15b(-); Punc-119*::*sid-1(+)* and *sid-1(-); nre-1(-)lin-15b(-); Pmec-3*::*sid-1(+)* fed with *mec-3* RNAi bacteria. Similarly, (H-I) Quaternary density in strains of similar genotypes fed with either *hpo-30* or *tiam-1* RNAi bacteria. The *Pmec-3*::*sid-1(+)* transgene is denoted as *PVD*::*sid-1(+)*. Statistics for G-I: One-way ANOVA with Tukey’s multiple comparison test, and number of worms (n), Biological replicates (N) are 24≤n≤47, N = 2, p<0.033*, 0.002**, 0.001***, ns, not significant.
(TIF)

S1 Table List of strains used in the study. Details of the strains with strain number, genotypes, and their sources are listed in this table.
(XLSX)

S2 Table List of RNAi bacteria used for the study. The source of RNAi bacteria and associated phenotypes are listed in this table.
(XLSX)

S3 Table Primers used in this study. List of primers and their sequences are mentioned in this table.
(XLSX)

S4 Table Details of statistical analysis. The mean, median, standard deviation, P values etc. are provided for various statistical analysis.
(XLSX)
