# Supplementary figures and images for: Optimization of RNAi efficiency in PVD neuron of C. elegans
Source: PLoS One. 2024 Mar 18;19(3):e0298766. doi: 10.1371/journal.pone.0298766 (PMC10947639; doi:10.1371/journal.pone.0298766)

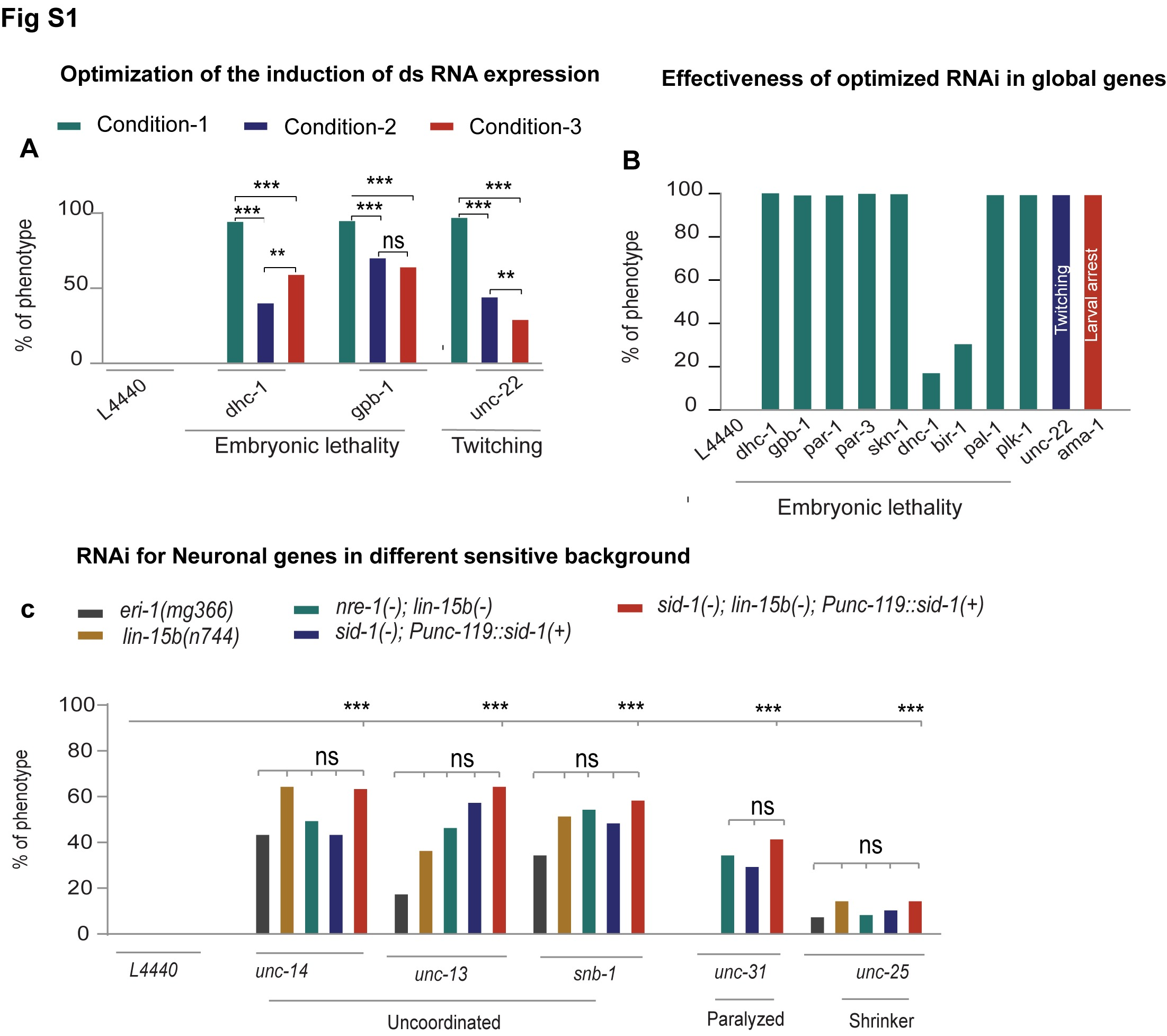

Supplement: S1 Fig — (A) The percentage of phenotype involving embryonic lethality and twitching in L4440 (control), dhc-1, gbp-1, unc-22 RNAi done in N2 Bristol background under three different induction conditions are plotted. Condition-I: induction of primary culture, Condition-II: induction of secondary culture with IPTG, and Condition-III: secondary culture was grown without IPTG induction. (B) The effectiveness of RNAi in N2 background using “condition-I” was further verified by knocking down various genes that cause embryonic lethality, twitching, larval arrest. (C) The organism-level phenotypes caused due to RNAi for genes required pan-neuronally are shown in this bar-plot. In this experiment, the RNAi was performed in the sensitive genetic backgrounds such as eri-1(mg366), lin-15b(n744), nre-1(hd20)lin-15b(hd126) and neuronal sensitive background sid-1(pk3321); Punc-119::sid-1(+) and sid-1(pk3321); lin-15b(n744); Punc-119::sid-1(+). (A-C) 10 P0s were fed with E. coli containing dsRNA and their progenies (300–350) were scored for respective phenotype in each batch. Biological replicates (1≤N≤2). (A-C) Statistics: Fisher’s exact test were performed, p<0.033*, 0.002**, 0.001***, ns, not significant. (TIF) [file pone.0298766.s001.tif]

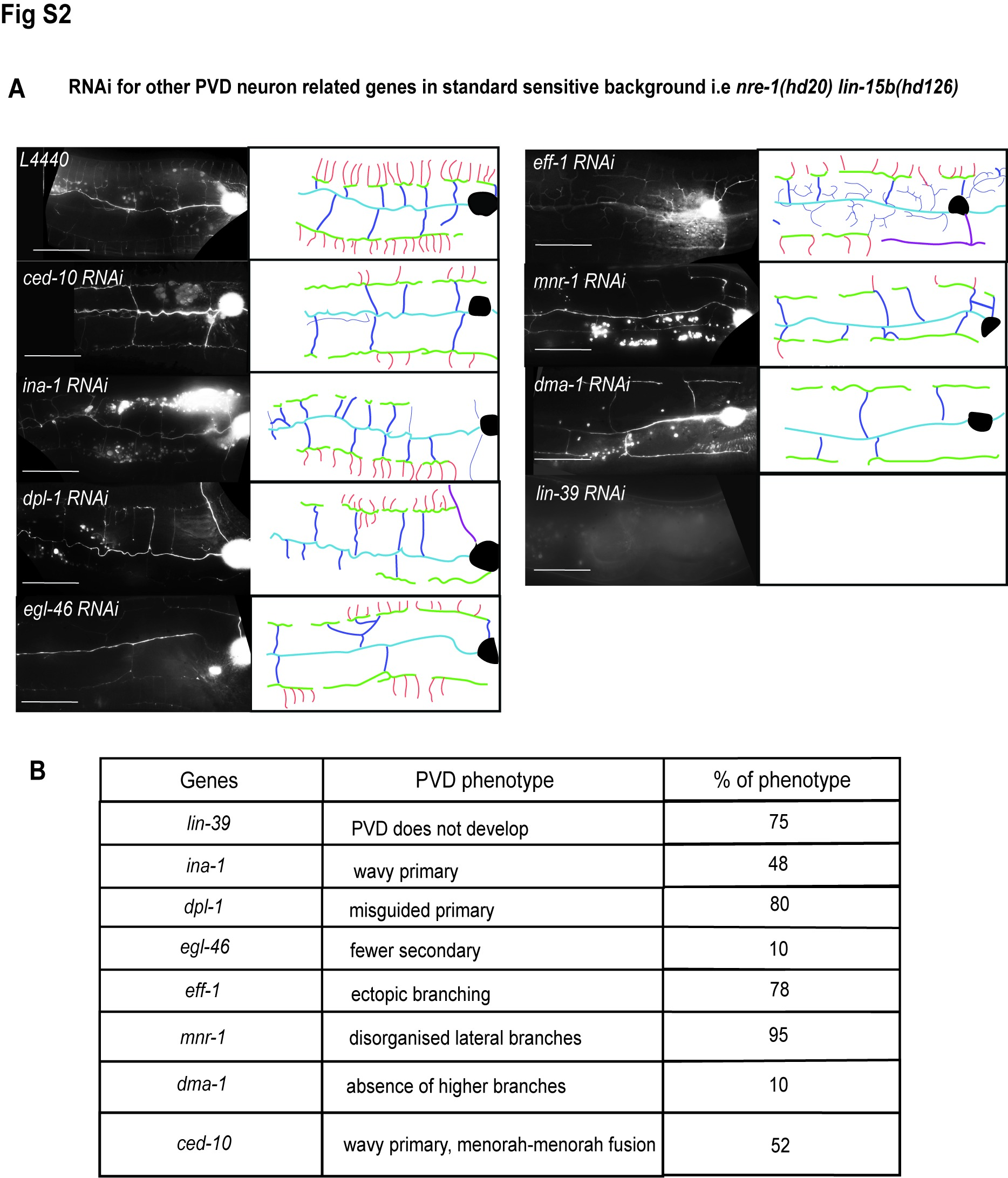

Supplement: S2 Fig — (A) The images show the dendrite morphology defects in PVD neuron caused due to RNAi of various genes known to affect PVD development. The RNAi of these genes were performed in nre-1(hd20)lin-15b(hd126) background. The illustrations of the defects caused due to RNAi of these genes are also shown on the right. The hierarchy of PVD dendrite are shown in different colors i.e quaternary in red, tertiary in green, secondary in violet, primary in blue. Scale bar is 25 μm. (B) The RNA experiment mentioned in panel-A is summarized in a tabular form. The phenotypes associated to the RNAi of various genes are mentioned in this table. Biological replicates (1≤ N≤2). (TIF) [file pone.0298766.s002.tif]

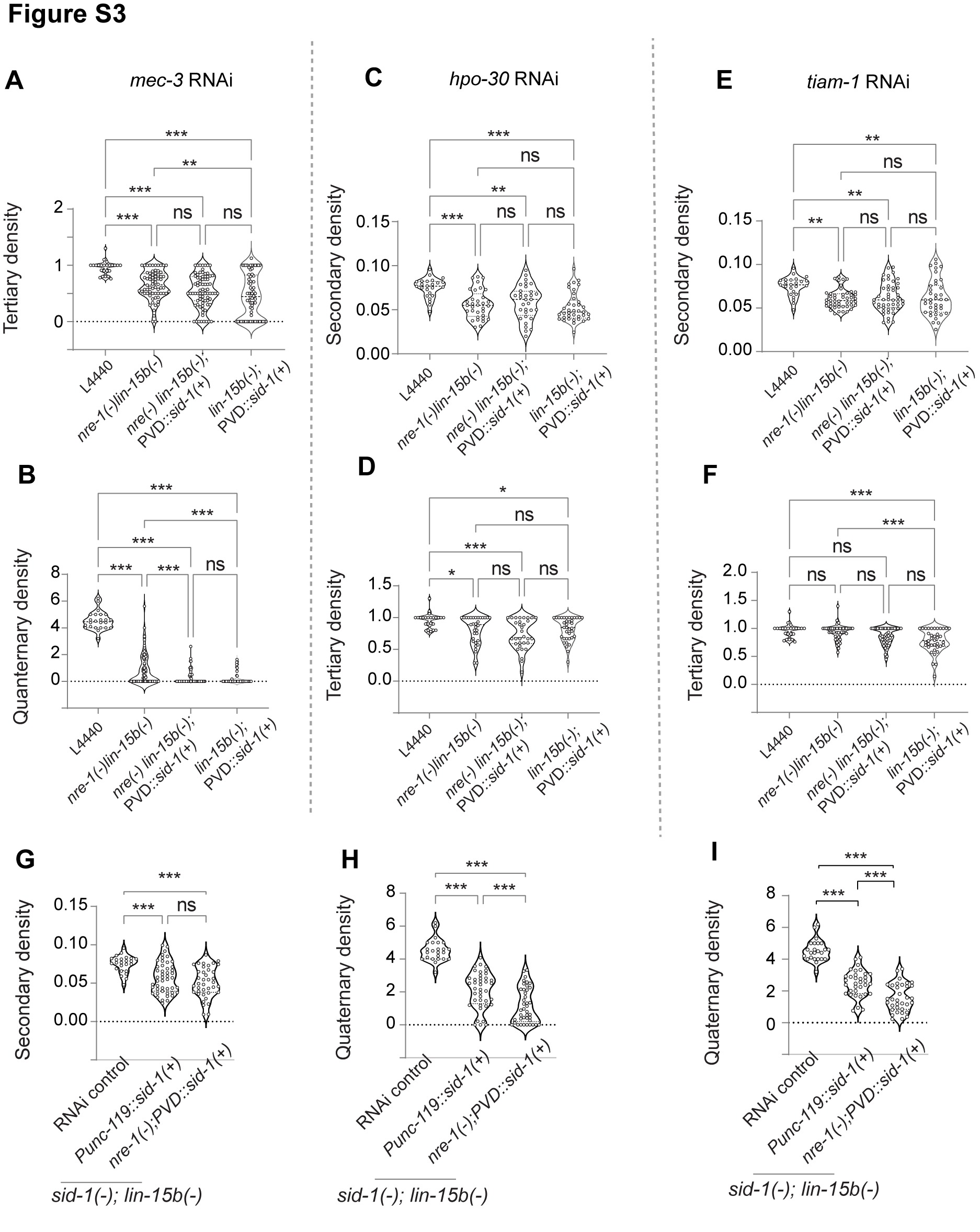

Supplement: S3 Fig — (A-B) Tertiary and quaternary density of PVD dendrites for mec-3 RNAi in different sensitive backgrounds. (C-D) represents secondary and tertiary density of PVD dendrites for hpo-30 RNAi worms. Similarly, (E-F) show the tertiary and secondary density for tiam-1 RNAi. Biological replicates (1≤N≤4) and number of worms (24≤n≤97). Statistics: One-way ANOVA with Tukey’s multiple comparison test p<0.033*, 0.002**, 0.001***, ns (not significant). (G-I) shows the comparative analysis of phenotypic penetrance involving PVD higher order branching in strains expressing sid-1 under unc-119 and mec-3 promoter. (G) shows the secondary density in strains i.e. sid-1(-); lin-15b(-); Punc-119::sid-1(+) and sid-1(-); nre-1(-)lin-15b(-); Pmec-3::sid-1(+) fed with mec-3 RNAi bacteria. Similarly, (H-I) Quaternary density in strains of similar genotypes fed with either hpo-30 or tiam-1 RNAi bacteria. The Pmec-3::sid-1(+) transgene is denoted as PVD::sid-1(+). Statistics for G-I: One-way ANOVA with Tukey’s multiple comparison test, and number of worms (n), Biological replicates (N) are 24≤n≤47, N = 2, p<0.033*, 0.002**, 0.001***, ns, not significant. (TIF) [file pone.0298766.s003.tif]
